# Maternal–Fetal Nutrient Transport in Pregnancy Pathologies: The Role of the Placenta

**DOI:** 10.3390/ijms150916153

**Published:** 2014-09-12

**Authors:** Kendra Elizabeth Brett, Zachary Michael Ferraro, Julien Yockell-Lelievre, Andrée Gruslin, Kristi Bree Adamo

**Affiliations:** 1 Healthy Active Living and Obesity Research Group, Children’s Hospital of Eastern Ontario Research Institute, 401 Smyth Rd., Ottawa, ON K1H 8L1, Canada; E-Mail: kebrett123@gmail.com; 2 Faculty of Health Sciences, School of Human Kinetics, University of Ottawa, 75 Laurier Avenue East, Ottawa, ON K1N 6N5, Canada; 3 Division of Maternal–Fetal Medicine, Obstetrics and Gynecology, the Ottawa Hospital, 501 Smyth Rd., Ottawa, ON K1H 8L6, Canada; E-Mail: zach.ferraro@gmail.com (Z.M.F.); 4 Ottawa Hospital Research Institute, Cancer Centre, 501 Smyth Rd., Ottawa, ON K1H 8L6, Canada; E-Mail: jyockell@ohri.ca; 5 Chronic Disease Program, Ottawa Hospital Research Institute, 501 Smyth Rd., Ottawa, ON K1H 8L6, Canada; 6 Faculty of Medicine, Pediatrics, University of Ottawa, 5 Laurier Avenue East, Ottawa, ON K1N 6N5, Canada

**Keywords:** placental transport, pregnancy, maternal, glucose, amino acids, fatty acids, fetal growth

## Abstract

Appropriate *in utero* growth is essential for offspring development and is a critical contributor to long-term health. Fetal growth is largely dictated by the availability of nutrients in maternal circulation and the ability of these nutrients to be transported into fetal circulation via the placenta. Substrate flux across placental gradients is dependent on the accessibility and activity of nutrient-specific transporters. Changes in the expression and activity of these transporters is implicated in cases of restricted and excessive fetal growth, and may represent a control mechanism by which fetal growth rate attempts to match availability of nutrients in maternal circulation. This review provides an overview of placenta nutrient transport with an emphasis on macro-nutrient transporters. It highlights the changes in expression and activity of these transporters associated with common pregnancy pathologies, including intrauterine growth restriction, macrosomia, diabetes and obesity, as well as the potential impact of maternal diet. Molecular signaling pathways linking maternal nutrient availability and placenta nutrient transport are discussed. How sexual dimorphism affects fetal growth strategies and the placenta’s response to an altered intrauterine environment is considered. Further knowledge in this area may be the first step in the development of targeted interventions to help optimize fetal growth.

## 1. Introduction

Pregnancy is a critical period of physiological change for both the mother and the fetus. As gestational age increases, so too does the need for energy to meet the nutritional demands of fetal development. Although in humans, only a modest increase of 340 and 450 kcal/day is required for the mother in the second and third trimester of pregnancy, respectively [1], maternal consumption must support her own basal metabolic function and continuously supply nutrients to the fetus. Pregnancy represents a natural state of maternal insulin resistance and the difference in maternal–fetal glucose concentration that increases with advancing gestation facilitates increased fetal macronutrient uptake [2]. Consequently, the metabolic needs of the growing fetus are met in part by the glucose concentration gradient across the maternal–fetal interface [3]. With advancing gestation, increases in fetal body weight are accompanied by changes in body composition such that there is a reduction in total body water concentration and large gains in white adipose tissue from the second trimester onwards [4,5]. The energy demands of fetal growth are substantial given the large caloric requirement associated with fat deposition, which accounts for 90% of energy deposited near term; the total estimated caloric requirement of a human fetus at term is 90–100 kcal/kg/day [6,7]. Energy intakes that diverge from the appropriate energy requirement may alter the fetal phenotype through epigenetic processes that alter expression of the genotype, such that insufficient or excess energy intake may cause growth restriction and overgrowth, respectively. Placental dysfunction can also restrict fetal growth by limiting nutrient supply to the fetus [8,9]. Intrauterine growth restricted (IUGR) fetuses are often born with depleted fat and glycogen stores [10,11]. In contrast, those born large-for-gestational-age (LGA), from mothers with obesity or to mothers who gain excessive weight during pregnancy, have increased adiposity [12,13,14] compared to average birth size newborns and mothers who gain the appropriate amount of weight, respectively.

In order to sustain appropriate fetal development the mother must provide glucose, amino acids and fatty acids, which are transported to the fetus across the placenta. There is increasing evidence that maternal factors, including body mass index, gestational weight gain, lifestyle behaviors (e.g., physical activity, smoking), as well as placenta-mediated diseases, can affect fetal growth and pregnancy outcomes. Although the precise mechanisms through which these factors affect fetal growth have yet to be fully elucidated, changes in placental nutrient transport to the fetus are implicated. This review provides an overview of placental nutrient transport, it explores how pregnancy-specific pathologies and maternal health behaviors may affect transporter expression and activity, and describes the molecular signaling pathways implicated in these changes.

## 2. Placenta Nutrient Transport

**Figure 1:**
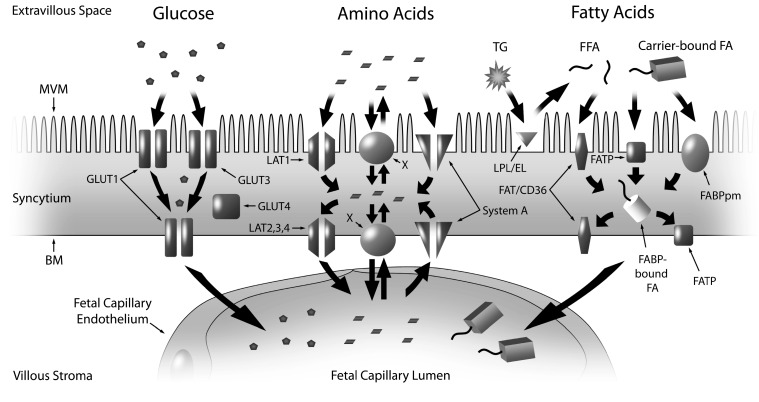
Nutrient transport across the placenta, featuring the SCTB and the fetal endothelium, and the location of key proteins involved in macronutrient (glucose, amino acids, fatty acids) transport at the MVM and BM. The SCTB is bathed in maternal blood on the apical surface instigating substrate transport at the MVM. This is followed by movement of the nutrients through the cytoplasm of the intermembrane space and interaction with the BM prior to uptake by the fetal capillary endothelium on the opposing side. Glucose is transported across the MVM and BM primarily by GLUT1. The accumulative transporters, System A, mediate the uptake of small neutral amino acids across the MVM and BM into the syncytium. Amino acids are transported across the BM towards the fetal capillary by System L facilitated transporters (TAT1, LAT2, 3 and 4) and exchangers. The exchangers, transport one amino acid in exchange for another, and thus they are dependent on the activity of the accumulative and facilitative transporters. LPL and EL hydrolyze maternal (TG) into FFA that cross the MVM through FATPs, FAT/CD36 and FABPpm. FFAs are trafficked through the cytosol via FABPs and across the BM by FATPs and FAT/CD36. Abbreviations: SCTB—syncytiotrophoblast; MVM—microvillous membrane; BM—basal membrane; GLUT—glucose transporter; LAT—large neutral amino acid transport; TG—triglycerides; LPL—lipoprotein lipase; EL—endothelial lipase; FFA—fatty acid; FAT/CD36—fatty acid translocase; FATP—fatty acid transport protein; FABP—fatty acid binding protein; FABPpm—plasma membrane fatty acid binding protein; X—exchangers.

Fetal growth is directly related to maternal nutrient availability and the placenta’s ability to transport these nutrients from maternal circulation to the fetus. The anatomical configuration of the placenta prevents direct contact of maternal and fetal blood, highlighting the importance of transport proteins, electrochemical gradients and diffusion channels for substrate exchange across the interface. Nutrient transport across the placenta and into fetal circulation is complex. There are two layers in the placental villi through which substrates, gases and water from maternal circulation must cross in order to reach the fetus [15,16]. The first layer, closest to maternal circulation, is made up of trophoblasts called syncytiotrophoblasts (SCTB), which line the villi. The SCTB constitute the transporting epithelium of the placenta, with two polarized membranes, the microvillous membrane (MVM) facing maternal circulation and the basal plasma membrane (BM) facing the fetal capillary. After passage across the SCTB membranes, substrates must cross the second layer of cells, the fetal capillary epithelium, before entry into the fetal circulation is complete (Figure 1). The fetal capillary endothelium is selectively permeable to molecules, such as amino acids and glucose, based on the size of the solute, and it is a relatively restrictive barrier against the diffusion of larger molecules [17,18]. Only smaller solutes are highly permeable through the MVM and BM, and thus the SCTB constitutes a barrier and rate-limiting step of the transport of nutrients into fetal circulation.

Complete maternal–fetal exchange across the SCTB relies on facilitated diffusion and active transport against concentration gradients to drive electrochemical potential and nutrient flux [19,20,21,22]. Consequently, the transport of nutrients and solutes across the SCTB occurs via a number of passive and active processes including flow-limited diffusion, transcellular diffusion, facilitated diffusion/protein-mediated transfer and endocytosis/exocytosis [21]. Nutrients predominantly enter fetal circulation through nutrient-specific transport proteins located within the MVM and BM. The types of transporter (e.g., facilitated, active, passive, uni- or bi-directional, *etc*.), the subtypes expressed in the placenta, and localization to the MVM and/or BM, have been thoroughly reviewed by others [23,24].

Placenta nutrient transport is dependent on placental size, morphology (exchange zone surface area and tissue thickness), nutrient transporter capacity/availability, and utero- and feto-placental blood flow [25,26]. With respect to placental size, placenta weight is a marker of the available surface area for maternal–fetal nutrient exchange. Placental weight is an important determinant of both birth weight and fetal growth [27], and fetal and placenta weight are positively correlated near term [28]. If the placenta fails to achieve an adequate size it may be unable to support the development of the fetus [29]. On the contrary, an association between large placentas and poor neonatal outcomes including hypoxia [29] and macrosomia [28] has also been reported. A marker of placental nutrient transporter efficiency is the fetal to placenta weight ratio (birth weight: placenta weight; in grams) [30]. This ratio can be altered by changes in placenta weight, fetal weight or both (reviewed by Fowden* et al.*) [31]. A lighter placenta and a higher fetal to placenta weight ratio is considered more efficient as it is consistent with the fetal drive to obtain nutrients from the placenta [31,32]. A lower fetal to placenta weight ratio may indicate below average placenta nutrient transport efficiency, and has been associated with increased pregnancy complications such as pre-eclampsia, c-section delivery and spontaneous pre-term delivery [28].

With regard to nutrient-specific transporters, the placenta’s capacity for nutrient transport can be altered by changes in the number, density, distribution or activity of these transporters [33,34,35]. Glucose, amino acids, free fatty acids (FFAs) and cholesterol are the essential macronutrients for adequate fetal growth, and each nutrient crosses the SCTB through specific transporters (Figure 1). Seminal work from Jansson and Powell has added experimental evidence to support the hypothesis that the placenta functions as a nutrient sensor [36]. Alterations in placental nutrient transport are thought to represent a control mechanism by which the fetal growth rate is matched with the availability of nutrients in maternal circulation—restricting growth when nutrition is limited and accelerating growth when nutrients are in excess [36]. To demonstrate, amino acid transport is down-regulated prior to the development of IUGR in rats fed a low protein diet [33,37], highlighting that maternal malnutrition can affect nutrient delivery and growth of the fetus. An alternate hypothesis suggests that the placenta may respond in a compensatory manner by up- or down-regulating transporter activity in response to low or high substrate levels, respectively, in an effort to maintain normal fetal growth. In normal pregnancies, smaller babies had higher amino acid transport activity [38], meanwhile, glucose transport activity was reduced in a hyperglycemic mouse model [39]. This “adaptive regulation” may serve to protect the placenta and fetus from under or excessive exposure to nutrients [40]. This review will focus on the first hypothesis, that the placenta functions as a nutrient sensor, and how it relates to common pregnancy pathologies. Placental nutrient transport phenotypes have been well-described in the context of IUGR and diabetic pregnancies [36] yet the proteins involved in nutrient transport are not sufficiently characterized, especially with respect to pregnancy complicated by obesity.

### 2.1. Glucose

Glucose is the primary energy substrate required for growth of the fetus and placenta. Fetal gluconeogenesis is minimal [41], and the fetus is almost entirely dependent on glucose from maternal circulation. Placental glucose transport occurs by facilitated diffusion along a concentration gradient through members of the glucose transporter (GLUT) family [3]. There are 12 members of the GLUT family, however GLUT1 is the only isoform abundantly expressed in early pregnancy and at term, and is the primary placental glucose transporter in humans [42]. There is an asymmetrical distribution of GLUT1 across the placental membrane, with a greater prevalence of GLUT1 on the MVM compared to the BM, suggesting that the rate limiting step of human placental glucose transport may occur at the BM [43]. Insulin like growth factor (IGF) 1, a known regulator of fetal growth [44], increases GLUT1 protein expression and glucose uptake at the BM but not the MVM [45]. GLUT3 and GLUT4 are present in first trimester placentas suggesting a possible role in glucose uptake early in pregnancy. GLUT3 is primarily localized at the MVM of the SCTB, although it is also expressed in the cytotrophoblast and endothelium [46]. GLUT3 expression decreases substantially in the second and third trimesters such that the level in the third trimester is only 34% of that observed in the first trimester [46]. The insulin-sensitive GLUT4 is localized in the cytosol of the SCTB [47], and at term the expression of GLUT4 is markedly reduced [47], suggesting a minimal role in glucose uptake from maternal circulation at term.

### 2.2. Amino Acids

Amino acids play a critical role in the development of fetal tissue. The plasma concentrations of most amino acids are higher in fetal circulation compared to maternal circulation [48], indicating active transport of amino acids across the SCTB [49]. The placenta expresses over 15 different amino acid transporters, and each is responsible for the uptake of several different amino acids [49]. The two most studied amino acid transport systems in the placenta are System A and System L [49]. System A is a sodium-dependent accumulative transport system which facilitates the transport of small neutral amino acids (SNAT) such as alanine, serine and glycine into the cell [49]. System A activity is present at both SCTB membranes, but is more highly expressed at the MVM [50]. The third trimester placenta expresses three isoforms of System A: SNAT1, SNAT2, and SNAT4 [51]. System A activity is stimulated by insulin, leptin, IGF1, and interleukin 6 [52,53,54]. System L is a sodium-independent exchanger for large neutral amino acid transport (LAT); it exchanges non-essential amino acids for predominantly essential amino acids with branched or bulky side chains, such as leucine [55]. System L is stimulated by glucose and insulin [54] and its activity depends on the activity of the other systems to provide the amino acids that drive the system L exchange function [55]. Different isoforms of system L are found on the MVM (LAT1) and the BM (LAT2, LAT3, LAT4) [56,57]. The rate limiting step in amino acid transport is believed to be across the MVM [58].The transport of amino acids across the BM into fetal circulation occurs via facilitated diffusion down their concentration gradients through the transporters LAT3, LAT4 and TAT1, as well as exchangers [56].

### 2.3. Fatty Acids

Fatty acids serve many critical roles in fetal growth including brain development and fat accretion. In maternal circulation, lipids are mainly found as triglycerides (TGs), phospholipids and cholesterol esters. TGs cannot cross the SCTB and are first broken down into FFAs by placental TG lipases [59]. The FFAs are then available for uptake into the placenta through FFA transport proteins [60]. Lipoprotein lipase (LPL) and endothelial lipase are both located at the MVM and hydrolyze TGs in the maternal circulation [61,62,63]. Endothelial lipase is also able to metabolize HDL, LDL and VLDL lipids [61,64]. The proteins associated with FFA transport include fatty acid transport proteins (FATP), fatty acid translocase (FAT/CD36), plasma membrane fatty acid binding protein (FABPpm), and fatty acid binding proteins (FABP).

FATPs are integral membrane proteins that are important for the uptake of long chain fatty acids [65]. There are six members of the FATP family, five of which have been identified in placental trophoblasts (FATP1–4, and 6) [66]. FATP1 and FATP4 are frequently studied in placental tissue as their expression correlates with docosahexanoic levels in maternal plasma, cord blood and placental phospholipids, suggesting an important role in the transfer of long chain polyunsaturated fatty acids [67]. The FATPs and FAT/CD36 are located on the MWM and BM and are involved in the transport of FFAs across the entire SCTB [68,69]. In contrast, FABPpm, which has a high affinity for long chain polyunsaturated fatty acids, is exclusively located on the MVM [68,69]. Five members of the FABP family (FABP1-5) have been identified in the trophoblast cells of the placenta and are localized in the cytoplasm of the SCTB [69]. The FABPs are responsible for cytosolic trafficking of FFAs to sites for esterification, beta-oxidation and subsequent transfer to the fetus [69]. The expression and activity of the proteins involved in fatty acid transport are influenced by insulin, IGF1 and leptin [70,71,72]. It remains unclear as to which step in the process limits the rate of placental fatty acid transport to the fetus.

### 2.4. Cholesterol/Lipoproteins

Cholesterol has an important role in fetal development, as it is an essential component of cell membranes and a precursor for steroid hormones. The fetus can synthesize cholesterol endogenously [73], but the placenta also transports cholesterol from maternal circulation to the fetus through cholesterol-carrying lipoproteins, such as low density lipoproteins (LDL), high density lipoproteins (HDL) and very low density lipoproteins (VLDL) [74]. The SCTB expresses lipoprotein specific receptors: LDL receptor (LDLR), scavenger receptor class B type I (SRBI) and VLDL receptor (VLDLR) [75,76,77]. Cholesterol from the placenta is transported to the fetus through specialized transporters, binding cassette transporter A1 and G1 (ABCA1 and ABCG1), located in the endothelial cells of the fetal vessels [78], as well as the MVM (ABCA1) and BM (ABCG1) [79,80].

## 3. Placental Nutrient Transport in Altered Fetal Growth

Growth restricted infants typically have poor neonatal outcomes, and thus the earliest work on placental nutrient transport focused on IUGR. This was followed by research on fetal overgrowth (e.g., macrosomia) in pregnancies complicated with diabetes. With the rise in maternal overweight and obesity, more recent work has focused on the impact of obesity on nutrient transport and fetal overgrowth (Table 1 and Table 2).

**Table 1:** Changes in expression level (protein or mRNA) and activity of the glucose, amino acid and fatty acid transporters in the human placenta associated with different pregnancy conditions ^1,2^.

Nutrient Transporter	IUGR—Placental Dysfunction	Type 1 Diabetes	GDM	Obesity
GLUT1	▬ [43,81]	▲* (BM) (birth weight > control) [82] ▬ (MVM) (birth weight > control) [82]	▬ (with and without LGA) [83]. ▲ (normal weight mothers; insulin controlled; no difference in birth weight) [84] ▬ (normal weight mothers; diet controlled; no difference in birth weight) [84] ▬ (obese mothers; diet or insulin controlled; no fetal overgrowth) [84]	▬ (no fetal over growth) [84]
GLUT3	▲ [85]			
GLUT4	▬ [85]		▼ (normal weight mothers; insulin controlled; no difference in birth weight) [84] ▼ mRNA (obese mothers; diet or insulin controlled; no fetal overgrowth) [84]	▼ mRNA (no fetal over growth) [84]
System A (SNAT1,2,4)	▼* (MVM) [58,86] ▬* (BM) [81]	▼* (MVM) (macrosomic) (no maternal BMI) [87] ▲* (MVM) (independent of fetal growth, similar maternal BMI) [88]	▲* (MVM) (independent of fetal over growth) [81]	▼* SNAT4 (no difference in birth weight) [89] ▬ SNAT1, SNAT2 (no difference in birth weight) [89] ▬* (no difference in birth weight) [90] * positive correlation to birth weight [90] ▲ SNAT2 (no difference in birth weight; positive correlation to birth weight) [90]
System L (LAT1-4)	▼* [91]		▼* (MVM) (fetal overgrowth) [81]	▬* (no difference in birth weight) [90]
LPL	▼* (preterm) [92] ▲ mRNA (preterm) [93]	▲* (macrosomic) [92] ▬ (macrosomic) [92] ▼ mRNA (birth weight > control; not macrosomic) [94]	▬ * ( macrosomic) [92] ▼ mRNA (birth weight > control; not macrosomic) [94]	▲* (no difference in birth weight) [95] ▬ mRNA (birth weight > control; not macrosomic) [96]
Endothelial Lipase	▲ mRNA (preterm) [93]	▲ (birth weight > control; not macrosomic) [97].	▬ (no fetal over growth) [98] ▲ (with obesity) (no fetal over growth) [98]	
FATP4				▼ (no difference in birth weight) [95] ▬ mRNA (birth weight > control; not macrosomic) [96]
FAT/CD36				▲ (no difference in birth weight) [95] ▼ mRNA (male) (birth weight > control; not macrosomic) [96] ▬ mRNA (female) (birth weight > control; not macrosomic) [96]
FABP1		▲ (macrosomic) [92]	▲ ( macrosomic) [92]	▼(no difference in birth weight) [95]
FABP3				▬ (no difference in birth weight) [99] ▼(no difference in birth weight) [95]
FABP4		▲ mRNA (birth weight > control; not macrosomic) [94]	▲ mRNA (birth weight > control; not macrosomic) [94]	▬ (no difference in birth weight) [99] ▲ (with diabetes) (birth weight > control; not macrosomic) [99] ▬ mRNA (birth weight > control; not macrosomic) [96]
FABP5			▲ mRNA (birth weight > control; not macrosomic) [94]	▲ mRNA (with diabetes) (birth weight > control; not macrosomic) [99] ▬ (no difference in birth weight) [99] ▼ mRNA (male) (birth weight > control; not macrosomic) [96] ▬ mRNA (female) (birth weight > control; not macrosomic) [96]
FABPpm				▬ (no difference in birth weight) [99] ▬ mRNA (birth weight > control; not macrosomic) [96]

^1^ Legend: ▬ no change in protein or mRNA expression, ▲ increase in protein expression (unless mRNA is indicated), ▼ decrease in protein expression (unless mRNA is indicated), * change in the activity of the transporter. If the box is left blank, there is currently no information on this transporter in this specific condition. Gender is specified only when a difference exists between the sexes; ^2^ IUGR—intrauterine growth restriction; GDM—gestational diabetes mellitus; GLUT—glucose transporter; SNAT—small neutral amino acid transporters; LAT—large neutral amino acid transporter; LPL—lipoprotein lipase; FATP—fatty acid transporter; FAT/CD36—fatty acid translocase; FABP—fatty acid binding protein; FABPpm—plasma membrane fatty acid binding protein.

**Table 2:** Changes in expression level (protein or mRNA) and activity of the glucose, amino acid and fatty acid transporters in the placenta in animal models of different pregnancy conditions ^1,2^.

Nutrient Transporter	IUGR—Nutrient Restriction	Maternal Diet
GLUT1	Mice §▼(no change in fetal weight) [100] Mice ¥▲(reduced fetal weight) [100] Sheep § ▲(reduced fetal weight) [101] Baboon ¥ ▼(reduced fetal weight) [102]	Mice ▲ (high fat) (increased fetal weight) [103]
GLUT3		Mice ▲ (high fat, high sugar) (§ reduced fetal weight; ¥ no change in fetal weight) [104]
System A (SNAT1, 2, 4)	Mice ¥ ▲SNAT1(reduced fetal weight) [100] Mice ¥ ▼ (reduced fetal weight) SNAT4 [100] Sheep § (reduced fetal weight) [101] Baboon ¥ ▼SNAT2 (reduced fetal weight) [102]	Mice ▲ SNAT2 (high fat) (increased fetal weight) [103] Mice (male) ▲ SNAT2 (“cafeteria” diet) (no change in fetal weight) [105] Mice (female) ▲ SNAT4 (“cafeteria” diet) (no change in fetal weight) [105] Mice ▲ SNAT2 (high fat, high sugar) (§ reduced fetal weight; ¥ no change in fetal weight) [104]
System L (LAT1–4)	Baboon ¥ ▼ LAT1/2 (reduced fetal weight) [102]	
FATP4	Sheep § ▲(reduced fetal weight) [101]	
FAT/CD36	Sheep § ▲ (reduced fetal weight) [101]	

^1^ Legend: ▬ no change in protein or mRNA expression, ▲increase in protein expression (unless mRNA is indicated), ▼ decrease in protein expression (unless mRNA is indicated), * change in the activity of the transporter, ¥ End of gestation in animal study, § Mid gestation in animal study. If the box is left blank, there is currently no information on this transporter in this specific condition; ^2^ IUGR—intrauterine growth restriction; GDM—gestational diabetes mellitus; GLUT—glucose transporter; SNAT—small neutral amino acid transporters; LAT—large neutral amino acid transporter; FATP—fatty acid transporter; FAT/CD36—fatty acid translocase.

### 3.1. Intrauterine Growth Restriction

IUGR is characterized by the fetus not reaching its predetermined growth potential and can result from a multitude of causes, including placental dysfunction and maternal under-nutrition. With placental dysfunction, the nutrient supply to the fetus is insufficient despite adequate nutrient availability in the mother. Insufficient remodeling of the spiral arteries during placentation is the key physiological change that contributes to inadequate blood flow to the fetus, resulting in a reduction in nutrient and oxygen delivery and IUGR [8,9]. In maternal under-nutrition, there may be inadequate food supply or deliberate calorie restriction and thus there is insufficient availability of nutrients in maternal circulation, often resulting in nutritionally-induced IUGR [106]. In a small subset of the population, maternal obesity can also increase the risk of IUGR [107,108,109]. However, the mechanisms involved in the development of IUGR in the context of maternal obesity are not well understood and lie outside the focus of this review.

#### 3.1.1. IUGR—Placental Dysfunction

With respect to glucose flux, although fetal hypoglycemia has been implicated in the pathophysiological mechanism of IUGR, this is not due to reduced glucose uptake or expression of the GLUT1 transporter at the SCTB [43,81]. However, there is increased expression of GLUT3 protein on the maternal aspect of the placenta in late-term IUGR compared to normal pregnancy, with no changes in GLUT1 or GLUT4 [85]. In this study, GLUT3 was expressed in the cytotrophoblast, and to a lesser degree in the SCTB, and it is thought that the increased expression of GLUT3 in the cytotrophoblast may contribute to an increased consumption of glucose by the placenta itself [85].

Placental amino acid transport in IUGR pregnancies has previously been reviewed [36,110,111], and reduced expression and activity of the amino acid transporters is consistently identified. This is intuitive, provided that amino acid concentrations in the cord blood in IUGR are significantly reduced compared to normal pregnancies [48]. Specifically, in IUGR the activity of System A is reduced in the MVM [58,86], but unaltered in the BM [81]. In preterm IUGR, System A activity in the MVM is reduced to a greater degree [81]. Additionally, the activity of the System L leucine transporter is reduced at the MVM and the BM [91], and taurine transporter activity is reduced at the MVM [112]. Taken together, diminished amino acid transporter activity, independent of altered expression, may be an adaptation by which the placenta responds to a suboptimal *milieu* in an attempt to regulate growth without compromising development of vital organs (brain and heart). Thus, down-regulation of amino acid transport capacity and efficiency to the fetus is likely an important contributing factor to the restricted fetal growth of these pregnancies.

In the case of fat transport, the activity of LPL was found to be decreased by 47% in preterm IUGR placentas, compared with preterm controls, with no differences observed in term IUGR [92]. On the contrary, Gauster* et al.* found that LPL mRNA expression was increased by greater than two-fold in preterm IUGR compared to normal term placentas, in conjunction with a 30% reduction in mRNA expression of endothelial lipase [93]. Altered lipoprotein receptor expression has also been identified in IUGR: an increase in LDLR protein and a reduction in SRBI protein compared to average-for-gestational-age controls [113]. These changes in TG hydrolases and lipoprotein receptors may contribute to the reduced adiposity that is typical in IUGR infants.

#### 3.1.2. IUGR—Maternal Nutrient Restriction—Evidence from Animal Models

Under-nutrition during pregnancy frequently occurs in developing countries and is also related to eating disorders, famine caused by natural disasters, food insecurity and voluntary calorie restriction to maintain a certain body image. Maternal nutrient restriction can alter placentation, and these effects may depend on the timing of the nutrient restriction. During the Dutch famine, babies who were exposed in mid to late gestation had less efficient placentas (birth weight adjusted for placenta area), in contrast, babies exposed in early gestation or who were conceived after the famine had ended had efficient placentas [114]. However, the influence of deliberate maternal nutrient restriction on placental nutrient transfer has only been investigated in animal models.

Using a murine model of nutrient restriction (80% of control diet), Coan *et al.* generated mice with reduced birth and placental weight as well as reduced fetal to placental weight ratio, compared to control mice, [100]. At Day 16 in the nutrient restricted mice, the gene expression of placental GLUT1 was reduced 83% to that of the controls, although, by Day 19, GLUT1 gene expression was significantly greater than in the controls suggesting an attempted compensatory response of a highly plastic system [100]. However, nutrient restriction did not alter unidirectional materno-fetal clearance of tracer glucose at either time point. There was no difference in amino acid transporter expression or activity at Day 16, however, at Day 19, nutrient restriction increased the gene expression of SNAT1 and decreased the gene expression of SNAT4, which corresponded with increased amino acid clearance [100]. Work by Ma* et al.* demonstrated that deliberate maternal nutrient restriction to 50% of the control diet during the first half of gestation could alter placenta nutrient transport in sheep [101]. At mid-gestation, protein and mRNA expression of GLUT1, FATP4 and FAT/CD36 were elevated in the placentomes of nutrient restricted ewes, likely to maintain placental efficiency and fetal weight, however, despite this adaptation, placental and fetal weights were reduced compared to controls [101]. In ewes that were nutrient restricted for the first half of pregnancy but were fed 100% of the control diet during the second half of pregnancy, only FATP4 mRNA and protein levels were increased compared to controls at the end of gestation, and there was no difference in fetal or placental weight [101].

Collectively, these studies demonstrate placental plasticity to adapt to maternal nutrient restriction by altering its phenotype in an effort to maintain normal fetal growth. However, changes such as increased transport of fatty acids and glucose have the potential to alter the fetal body composition which could continue to impact the offspring in postnatal life [115]. Recently, Kavitha* et al.* were the first to explore changes in placenta nutrient transporter expression in response to maternal nutrient restriction in a non-human primate [102]. Maternal nutrient restriction during gestation (70% of control diet) in baboons reduced fetal weight, as well as the MVM protein expression of GLUT1 and the amino acid transporters TAUT, SNAT2, and LAT1/2 compared to the controls [102]. Given the similarities between human and primate reproductive physiology and placenta structure, this study is highly relevant for human health, nutrient deprivation and altered fetal growth, as it is not ethical to subject pregnant women to experimental nutrient restriction.

### 3.2. Fetal Overgrowth

Although not always the case, pathologies such as diabetes and obesity may result from positive energy balance, a net surplus of hormones (e.g., insulin), and/or growth dysregulated factors (e.g., IGF) [116,117], thereby increasing the risk of fetal overgrowth. Diabetes during pregnancy is associated with maternal hyperglycemia and hyperlipidemia, and thus increased glucose supply and altered lipid delivery to the fetus [118,119], whereas obesity during pregnancy is associated with elevated maternal lipid levels [59]. Obese mothers, and those who gain excess gestational weight, tend to birth infants with increased neonatal fat mass and body fat percentage [12,13,120], providing evidence that maternal adiposity predicts neonatal fat mass and not simply overall mass**.** It is thought that the increased availability of nutrients in maternal circulation stimulates the placenta to increase transport of these nutrients thus resulting in fetal overgrowth.

#### 3.2.1. Diabetes

In pregnancies complicated by maternal diabetes, the alterations in nutrient transport differ between type 1 diabetes and GDM, and this has been extensively studied and documented in the seminal work by Jansson and Powell and colleagues [83,88,92]. In type 1 diabetes, studies show altered System A activity. For instance, System A activity was reduced by 49% in the MVM in macrosomic infants born to women with type 1 diabetes [87], in contrast, an increase in System A activity by 65%–80% has also been reported in type 1 diabetes, independent of fetal over-growth [88]. Of note, maternal weight was not accounted for in the former study [87], but maternal body weight was similar across groups in the latter [88], and thus the pathology of the diabetes in the mother may be different across studies affecting the results. Type 1 diabetes is associated with increased GLUT1 expression and increased glucose uptake at the BM, compared to healthy pregnancies, but no alterations were found at the MVM [82]. Compared to appropriate-for-gestational-age infants born to healthy mothers, type 1 diabetes was also associated with an increase in LPL activity (but no difference in protein expression), an increase in FABP1 protein expression [92], and an increased expression of endothelial lipase [97]. Data gathered through microarray profiling of placenta samples has indicated that type 1 diabetes is associated with a 2.4-fold up-regulation of FABP4 compared to control, however, LPL expression was down-regulated nearly 3.4-fold [94]. The difference in LPL expression could be related to the fetal outcomes or how LPL was measured; Magnusson* et al.* observed higher LPL activity with macrosomic infants (but no change in protein expression) [92], conversely, Radaelli* et al.* found that LPL expression was down-regulated, but the infants were not macrosomic [94]. These inconsistencies highlight the importance of comparing homogeneous populations (*i.e.*, accounting for maternal and fetal characteristics), as well as exploring all aspects of expression (gene, protein, activity).

The work conducted in GDM pregnancies is more difficult to interpret than type 1 diabetes, as treatment is not consistent across patients. Some women regulate their GDM with diet and exercise alone, while other women are required to take insulin. In most studies examining placenta nutrient transport characteristics, the mode of diabetes treatment is not considered and the groups contain both diet and insulin treated women, which might influence the pathology. In an earlier study of GDM pregnancies (22% insulin treated; 78% diet controlled; no record of maternal BMI), it was found that compared to pregnancies not complicated by diabetes, the expression and activity of GLUT1 was unaltered [83]. However, in studies that accounted for treatment modality and maternal BMI, the results differed according to treatment and BMI. In non-obese women, those with insulin controlled-GDM had higher protein and mRNA expression of GLUT1 when compared to non-obese diet controlled-GDM and healthy controls (protein only), as well as lower protein and mRNA expression of GLUT4 when compared to non-obese diet controlled-GDM (mRNA only) and healthy controls [84]. Furthermore, in the obese women with insulin controlled-GDM, GLUT4 mRNA expression was less than that of obese women with diet controlled-GDM and the obese, non-diabetic controls, but there was no change in protein expression [84]. Thus demonstrating the importance of considering maternal BMI and the manner in which the GDM is treated.

With regards to amino acid transport, System A activity was higher at the MVM in women with GDM, with and without LGA babies, when compared to healthy controls, whereas amino acid transport was unaltered in cases of fetal over-growth alone [81]. This suggests that the change in System A activity is a response to the diabetic environment and not a feature of fetal over-growth. Additionally, we can infer that the type of treatment for GDM likely does not influence the pathology, as only seven women with GDM were treated with insulin (1/10 in the GDM group, 6/10 in GDM/LGA group), yet both groups exhibited similar levels of System A activity. System L activity was also higher at the MVM in GDM pregnancies with fetal over-growth [88]. In obese women with GDM (dietary regimen or insulin therapy), endothelial lipase expression was greater, however, obesity or GDM (dietary regimen or insulin therapy) alone had no effect on its expression [98]. In one study, the protein expression of FABP1 was 64% higher in GDM (insulin therapy (*n* = 3), diet only (*n* = 5)) when compared to appropriate-for-gestational-age infants born to healthy women, however, the activity of LPL was unaltered [92]. Using microarray profiling, GDM (all insulin therapy) preferentially activated genes related to lipid metabolism, with a two-fold up-regulation of FABP4 and FABP5 compared to control, however, LPL expression was down-regulated nearly three-fold [94]. In placental explants of women with GDM (all insulin therapy), fatty acid oxidation was reduced by 30%, and the TG accumulation was three-fold higher* vs.* non-diabetic control [121]. These alterations in placental lipid metabolism are thought to be a regulatory step contributing to the fetal fat accumulation and macrosomia that often accompanies GDM.

Gestational diabetes mellitus can also alter the expression of placental cholesterol transport proteins. At the SCTB, the LDLR mRNA and protein expression was significantly higher in women with GDM (all insulin therapy), independent of their BMI, compared to healthy controls. VLDLR expression was higher in overweight/obese women with GDM compared to the normal weight women with GDM and the healthy controls, and SRBI expression was significantly higher in normal weight women with GDM compared to the other groups [118].The expression of ABCA1 was significantly lower in women with GDM independent of BMI, meanwhile, the expression of ABCG1 was significantly lower only in overweight/obese women with GDM [118]. On the whole, these findings suggest that maternal diabetes may disrupt normal placenta nutrient transporter expression and activity, which may contribute to the accelerated fetal growth in these pregnancies.

#### 3.2.2. Obesity

There is limited research on the effects of maternal obesity on placenta nutrient transport, particularly in normoglycemic humans. To our knowledge, there is only one study examining the impact of obesity on glucose transporters in the human term placenta. This study found no difference in GLUT1 mRNA or protein expression and only a significantly lower GLUT4 mRNA but not protein expression in the obese, normoglycemic patients, compared to healthy weight, normoglycemic patients [84]. However, there was no difference in birth weight between groups, raising the possibility that differences may not be present due to the similar fetal outcomes. Similar transport dynamics were also explored using high fat fed mice as a model of obesity [103]. Compared to the control mice, the high fat fed mice had increased fetal weight, an increased rate of glucose clearance and increased GLUT1 expression in the placenta [103]. This discrepancy between rodent and human models highlights the importance of considering whether the human subjects fully represent the population you aim to study. For instance, in this case, did the obese women with healthy weight children truly represent the obese phenotype that the authors wished to study?

The impact of obesity has been explored on placental amino acid transport in humans, and decreased activity and expression of SNAT4 was shown in obese women compared to lean women, despite no difference in infant birth weight, meanwhile, SNAT1 and SNAT2 expression was unchanged [89]. These results were contrary to the author’s hypothesis and raised the possibility that reduced amino acid transfer and increased transport of FFA or glucose across the placenta (not measured) might occur in the obese women. We theorize that this could result in offspring of similar weight in the obese and lean women, but with increased adiposity and lower lean mass in the infants from obese mothers [13]. At term, the contribution of SNAT4 to amino acid transport is reduced, while SNAT1 and SNAT2 are believed to be the predominant contributors to System A transport [51,122]. Thus is it interesting that SNAT activity was decreased in the obese women despite similar expression of the key System A transporters at term. It is possible that the similar expression of SNAT1 and SNAT2 may have contributed to the similarities in birth weight between groups [89]. Conversely, recent work in humans found no difference in the activity of System A or System L transporters when comparing normal weight to overweight/obese women (with no differences in birth weight), however, MVM System A activity was positively correlated with birth weight, but not maternal BMI [90]. Furthermore, the expression of SNAT2 was positively correlated with maternal BMI and birth weight, but neither SNAT1 or SNAT4 were correlated to birth weight or BMI [90]. Given that System A activity was positively correlated with birth weight, it is possible that differences between groups might have been observed had the women with obesity given birth to macrosomic infants**.** Despite the contradictory results there is some evidence to suggest a possible link between maternal obesity, altered amino acid transport and increased fetal growth. Further work should consider using a more concise definition of the phenotype associated with pregnancies complicated by obesity (*i.e.*, high maternal BMI, LGA fetus) to better understand the underlying mechanisms linking maternal habitus to fetal size at birth.

There is a paucity of research on the impact of maternal obesity and the expression or activity of FFA transport proteins in human pregnancies, although evidence from an obese ovine model suggests that obesity alters placental fatty acid transport through changes in transporter levels and not TG hydrolysis. In this study of sheep, Zhu *et al.* found elevated fetal blood levels of cholesterol and TG, and increased FATP1 and FATP4 protein levels, but no difference in LPL expression [123]. In 2011, Scifres* et al.* found that placentas from obese-diabetic women exhibited an increase in FABP4 and FABP5 mRNA, and FABP4 protein expression, but no change in FABP3 or FABPpm, when compared to obese, non-diabetic or normal weight women [99]. However there were no differences between the obese non-diabetic and the normal weight women [99]. Furthermore, in 2012 Dubé* et al.* found that maternal obesity was associated with significantly higher FAT/CD36 mRNA and protein levels and LPL activity, but a lower expression of FATP4, FABP1 and FABP3 protein compared to the normal weight women [95]. However, the most recent evidence suggests that the susceptibility of the placenta to maternal factors may be fetal sex specific, and it may be important to explore outcomes in a sex specific fashion [96,124,125]. In particular, Brass* et al.* found the rate of placental oleic acid uptake was 43% lower in male offspring and 73% higher in female offspring born to obese women, compared to lean women [96]. Changes in placenta mRNA levels were also dependent on fetal sex, with lower FAT/CD36 and FABP5 expression among male offspring from obese women, whereas gene expression levels were unchanged in female placentas, regardless of maternal BMI [96]. This study also found no detectable differences in placental gene expression of LPL, FATP4, FABP4 and FABPpm between groups [96]. However, in the first two studies there was no difference in birth weight in the infants born to the lean and obese women [95,99]), and in the latter study, the infants born to the women with obesity were heavier than the infants born to the lean women, but they were not macrosomic [96]. It is possible that greater changes in transporter expression and activity would have been observed had the impact of the maternal obesity been more apparent on the child (*i.e.*, macrosomia)**.** Given the paucity of work in this area and the inconsistent results, it remains unclear if obesity is associated with specific alterations in placental nutrient transport and it is evident that further work is needed examining the sex-specific differences.

## 4. The Impact of Maternal Diet

The high incidence of obesity and metabolic diseases is largely attributed to our unhealthy modern food environment, which promotes high calorie, but low nutrient quality diets. Given that fetal growth is closely related to the maternal nutrient supply, it may be important to explore the impact of unhealthy, excessive and unbalanced dietary composition on placental nutrient transport. Preliminary work has explored this topic in animal models. In a mouse model, the *ad libitum* consumption of a high fat diet (32% fat, 52% carbohydrate, 16% protein) for 8 weeks before mating and during gestation increased placenta transport of glucose (5-fold) and neutral amino acids (10-fold), and increased the expression of GLUT1 (five-fold) and SNAT2 (9-fold) [103]. In another mouse model, *ad libitum* access to a high fat, high sucrose “cafeteria diet” (58% fat, 25.5% sucrose, 16.4% protein) increased the expression of SNAT2 in male placentas and SNAT4 in female placentas [105]. Additionally, a high sugar, high fat diet (30% fat, 17% protein, 53% carbohydrate) during pregnancy in mice reduced placenta weight, increased placenta glucose and amino acid transport, and increased the expression of GLUT3 and SNAT2 [104]. Diets with varying compositions of fat and fiber have also altered the placenta mRNA expression of GLUT1, GLUT3 and SNAT4 in mice [126]. Although only explored in rodent models, it is important to appreciate that the absolute caloric quantity in addition to maternal diet composition (*i.e.*, diet quality) may play an important role in altering placenta nutrient transport to the fetus.

## 5. Molecular Mechanisms Regulating Altered Nutrient Transport

The molecular mechanisms responsible for alterations in placental nutrient transport are largely understood to involve the mammalian target of rapamycin (mTOR) signaling pathway. The placenta nutrient sensing model, proposed by Jansson and Powell, suggests that mTOR, located in the trophoblasts cells, serves as the integrator of signals from maternal supply and fetal demand [20,127]. The mTOR pathway integrates various signals to regulate growth, including growth factors, stress, energy status, oxygen and amino acids [128]. mTOR receives maternal signals (e.g., insulin, leptin) at the MVM and transmits this information downstream to alter gene transcription and protein translation, resulting in up/down regulation of nutrient transport proteins implicated in fetal growth. The positive regulators of mTOR include the maternal hormones insulin, leptin and IGF1 [52,54,129,130], while adiponectin and hypoxia inhibit mTOR [129,131,132]. In particular, in cultured primary human trophoblast cells, the stimulation of System A activity by insulin and IGF1 is dependent on mTOR signaling [54]. Pregnancy complications, such as obesity, that alter maternal nutrient supply, adipokine, cytokine and hormone levels can alter the mTOR pathway, thus leading to modifications in placenta nutrient transport and consequently modify fetal growth.

**Figure 2:**
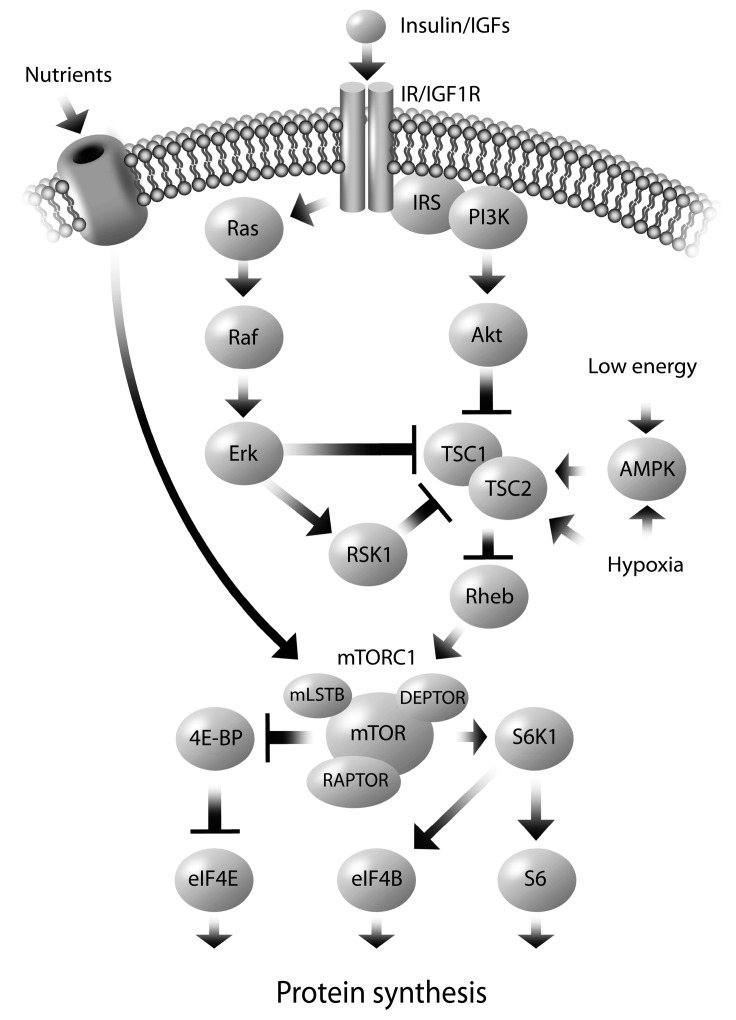
Regulation of the mTORC1. Various upstream kinases (Akt/PI3K, ERK1/2, RSK1) converge on TSC1/2, which regulates mTOR through Rheb. Activation of mTORC1 leads to the phosphorylation of S6K and the dissociation of eIF4E from 4E-BP, which in turn promotes protein synthesis. Insulin/ IGF phosphorylates Akt, which inhibits TSC2, thus releasing the inhibition of Rheb by TSC1/2. Activated Rheb stimulates mTORC1 signaling. AMPK, in response to low energy levels or hypoxia, phosphorylates TSC2, and thus inhibits mTORC1. Nutrients, specifically amino acids, activate mTORC1, independently of TSC1/2. Abbreviations: mTOR—mammalian target of rapamycin; mTORC1—mTOR complex 1; TSC—tuberous sclerosis complex; Akt/PKB—protein kinase B, ERK—extracellular-signal-regulated kinase, RSK1—MAPK-activated, p90 ribosomal S6 kinase 1; IGF—insulin like growth factor; IRS/PI3K—insulin receptor substrate/phosphoinositide 3-kinase; AMPK—AMP activated kinase; S6K1—p70 ribosomal S6 kinase 1; 4EBP1—eukaryotic initiation factor 4E-binding protein; eIF4E—eukaryotic initiation factor 4E; eIF4B—eukaryotic initiation factor 4B; S6—ribosomal protein S6

mTOR is an atypical serine/threonine protein kinase that interacts with several proteins to form two distinct complexes named mTOR complex 1 (mTORC1) and 2 (mTORC2); albeit mTORC1 is better characterized in placenta nutrient sensing (Figure 2). mTOR is controlled by the intermediate Rheb which is regulated by the Tuberous Sclerosis Complex (TSC1/2). Various upstream effector kinases, such as protein kinase B (Akt/PKB), extracellular-signal-regulated kinase 1/2 (ERK1/2), and MAPK-activated, p90 ribosomal S6 kinase 1(RSK1) act on the TSC1/2 complex, thus affecting mTOR activity [128]. It is through these pathways that TSC1/2 transmits many of the upstream signals that act on mTOR, including growth factors and cytokines. For instance, growth factors, such as insulin and IGF1, signal through the IRS/PI3K and Akt/PKB pathway, which inactivates TSC1/2, thus activating mTORC1 in the presence of anabolic, growth promoting, signals. When activated, the ERK1/2 and RSK1 pathways have been shown to inhibit TSC1/2, thus activating mTOR. Pro-inflammatory cytokines, such as TNFα, may also lead to activation of mTORC1 through a mechanism similar to that of the growth factors by phosphorylating and inhibiting TSC1/2. Although, the mechanism is not fully elucidated, amino acids appear to activate mTORC1 independently of TSC1/2 [128]. mTOR also engages in extensive cross-talk with AMP activated kinase (AMPK) in response to cellular stress and energy depletion [133,134]. AMPK phosphorylation (activation) occurs via an accumulation of AMP and hence detects energy depletion (low ATP levels) which inhibits mTOR [133,135].

Protein synthesis is the best-characterized process that is mediated by mTORC1 activation. In primary villous explants and cultured primary human trophoblasts, mTOR is a positive regulator of System A and System L amino acid transporters [136,137]. This occurs predominantly at the post translational level by altering the transporter abundance at the plasma membrane through mTOR activation [138]; which may increase the cell surface abundance of amino acid transporters. The downstream proteins directly phosphorylated by mTORC1 are p70 ribosomal S6 kinase 1 (S6K1) and the eukaryotic initiation factor 4E-binding protein 1 (4EBP1), regulate translation [128]. The phosphorylation status of S6K1 and 4EBP1 are used as an index of mTORC1 activity. Amino acid deprivation inhibits the activity of mTORC1, evident by the dephosphorylation (inactivation) of S6K1 and 4EBP1 [139].

Alterations in the mTORC1 signaling pathway have been identified in pregnancies associated with abnormal fetal growth. Although substantial mechanistic evidence is available from* in vitro* work using trophoblast and BeWo cell line models of placenta physiology [136,137,138,140], we have focused on evidence from animal and human studies. When maternal nutrient availability is restricted, the activity of placental mTOR is decreased, such as in human IUGR [136,141], in protein restriction in the rat [33,142] and in nutrient restricted baboons [102]. Specifically, in the baboon at the end of gestation, maternal nutrient restriction is associated with decreased phosphorylation of the upstream (IRS1, Akt, ERK1/2, RSK1) and downstream (4EBP1, S6K1) proteins in the mTOR signaling pathway, as well as reduced placental expression of glucose and amino acid transporters and reduced fetal weights [102]. The down-regulation of mTOR signaling and nutrient transport in response to maternal nutrient restriction suggests that the placenta is matching fetal growth with the availability of nutrients, such that the offspring is smaller and thus a better match for an environment with limited nutritional resources. In nutrient restricted sheep at mid-gestation, the activity of placental AMPK and ERK1/2 was increased, as were GLUT1, FATP4, and FAT/CD36 protein levels, and fetal weight was reduced, however, the activity of mTOR and Akt signaling were not altered [101]. mTOR is activated by ERK1/2, but inhibited by AMPK, and these conflicting signals may contribute to the similarities between groups in mTOR activity. After re-alimentation to the control diet, the nutrient restricted fetuses reached similar weights to the control group at the end of gestation [101], although these offspring had greater adiposity and reduced insulin sensitivity [115]. This suggests that in certain circumstances where nutrients are restricted, that the placenta stimulates several mechanisms which may act independent of the mTOR pathway, in an effort to augment nutrient transport (*i.e.*, glucose and fatty acids) to optimize fetal growth in less than favorable environmental conditions.

When maternal nutrients are in excess, the mTORC1 signaling pathway is activated, as demonstrated in large for gestational age (LGA) babies born to obese women [90], and in high fat fed overweight rats [143]. In the obese women who gave birth to LGA babies, the activity of AMPK (which inhibits mTORC1), was decreased likely due to an excess of nutrients, and the insulin/IGF1 signaling pathway (which activates mTORC1) was activated, in association with increasing BMI and birth weight [90]. Additionally, the phosphorylation of the downstream targets of mTORC1 (S6K1 and 4EBP1) were positively correlated to early pregnancy BMI and birth weight [90]. This suggests that up-regulation of the mTOR signaling pathway with increasing maternal BMI may contribute to the increased amino acid transport and birth weight of these LGA babies [90]. In contrast, in obese, over-nourished sheep at mid-gestation, there was a reduction in total and phosphorylated AMPK, as well as reductions in total mTOR and ERK1/2, and phosphorylated Akt, mTOR and ERK1/2 [144], with no difference in fetal weight at the end of gestation [123]. Overall, an excess of nutrients might inhibit the mTOR pathway, potentially through a negative feedback loop [145], in an effort to restrict excessive nutrient transport to optimize fetal growth. To illustrate, an excess of nutrients might result in continuous activation of mTOR-S6K1 signaling, which induces a negative feedback loop to attenuate PI3K signaling by inhibiting IRS [145]. Collectively, this evidence suggests that dysregulated placental mTOR is implicated in abnormal fetal growth and that a complex interplay between maternal nutrient status and fetal growth is tightly regulated through molecular mediators that may be altered in human pregnancy pathologies.

## 6. Sex Dependent Regulation of Fetal Programming

A discussion about the regulators of fetal growth would be incomplete without considering fetal sex. In 2007, a review by Di Renzo and colleagues identified male sex as an independent risk factor for adverse pregnancy outcomes, including a higher rate of preterm birth, gestational diabetes mellitus (GDM), and macrosomia, with evidence suggesting that females have a better outcome in the perinatal period, particularly after preterm birth [146]. In 2010, David Barker’s lab proposed that “boys live dangerously in the womb”, due to their risky growth strategy, characterized by a quicker rate of fetal growth with less investment in placental growth, thus increasing their vulnerability to under-nutrition [147]. These differences in growth and survival suggest that male and female fetuses may not have identical responses to intrauterine stressors, and evidence suggests that this differential susceptibility to fetal programming insults is potentially mediated by sex specific differences in the placenta. In fact, adverse conditions in pregnancy appear to have a sexually dimorphic effect on fetal outcomes, with greater placental adaptation observed in female offspring [148]. Indeed, the Dutch Famine is associated with changes in placental size and later risk for hypertension in men, but not in women [149]. Sexual dimorphism in the human placenta has been noted in cytokine expression, the insulin-like growth factor pathways, and the response to cortisol in relation to asthma during pregnancy (reviewed by Clifton 2010) [124], and recently in preeclampsia, with significantly higher pro-inflammatory cytokine production and apoptosis in the male placentas [125]. In rabbits, a high fat diet compared to a control diet during gestation induced sex specific adaptations in the placenta, including fatty acid accumulation in the female placenta thus protecting the fetus from dyslipidemia, and a down-regulation of the gene LXRα (liver X receptor; involved in cholesterol exchange) in male placentas [150]. In a primate model (baboons), maternal nutrient restriction during gestation did not result in a synchronized molecular response in the placenta when fetal sex was not accounted for. However, when the sexes were treated as separate groups, the female placentas exhibited a highly coordinated response to the nutrient restriction which was absent in the males [151]. This adaptive response of the female placenta was also recently observed in humans. Walker and colleagues found a strong negative correlation between gestational weight gain and placental glucose uptake in the female placentas, but no significant relationship was observed in the males [152], suggesting that the female placentas were able to adapt to the excess gestational weight gain in order to optimize glucose supply to the fetus. It has been proposed that sex-specific placental adaptations attempt to cope with the same adverse maternal environment, thus underlying the importance of considering fetal sex when designing and analyzing placental tissue experiments [124].

## 7. Conclusions and Future Directions

There are immediate and long-term health consequences associated with fetal under- and over-growth. The placenta plays a pivotal role in offspring growth and adequate nutrient transport is critical to support this development. A thorough understanding of nutrient transport is vital to elucidating the mechanisms contributing to altered fetal growth. In addition, having a greater understanding of placenta metabolism of glucose, lipids and amino acids with respect to obesity, excessive gestational weight gain, GDM and fetal overgrowth is an important area of study. While some pregnancy complications and nutrient transporters have been extensively studied, much of the research remains inconclusive regarding how certain transporters (*i.e.*, fatty acid transporters) are altered as a result of common pathologies (e.g., obesity). There are inconsistencies in the research findings concerning mechanisms of placenta transport in response to metabolic pathologies. We believe that these discrepant research findings are largely due to the heterogeneous methodology and populations explored (*i.e.*, the populations are not properly characterized).

With respect to GDM, the mode of treatment (diet controlled* vs.* insulin controlled) is an important consideration as the treatment likely influences the maternal glycemic control, and thus influences the severity of the insult to the intrauterine environment. For instance, as previously mentioned, in normal weight mothers with GDM, treatment affected the expression of GLUT1 [84]. Future studies in GDM pregnancies should consider the independent effect of diet and pharmacological (insulin or metformin) control of blood glucose, as different treatments modalities may influence the pathology. Moreover, it is also important to consider maternal BMI as the combination of GDM and obesity might alter the response [99]. Similarly, fetal birth weight is a crucial consideration when studying pregnancy pathologies that are related to fetal overgrowth (*i.e.*, diabetes and maternal obesity). For instance, if there is no difference in birth weight between the infants born to the lean and obese women, than it is important to consider the sample size of the population to ensure sufficient power to detect changes in addition to assessing for potential confounders including maternal behaviors (diet and physical activity) and clinical parameters such as severity of impaired glycemia and dyslipidemia. It is possible that if there are insufficient differences in the fetal outcomes (*i.e.*, birth weight), then there may not have been substantial variation in nutrient transport across the placenta. To appropriately identify contributors to altered fetal growth, it is likely necessary that differences in fetal growth exist between the populations being compared. In pregnancies complicated by maternal obesity, selecting only those with fetal macrosomia may be the ideal method to compare an obese to a lean pregnancy. Ensuring that the subject groups are sufficiently different with no other underlying pathology will ensure as representative a human model as possible.

Additionally, recent evidence suggests that male and female placentas might respond in different ways to an adverse intrauterine environment, and thus future research must consider sex-related differences of the infant when exploring changes in the placenta to avoid masking potential important findings. It has been proposed that ignoring the sex of the placenta is no longer sound scientific practice [124] as one cannot assume that the male and female fetus and placenta respond to environmental and maternal insults in the same manner.

Furthermore, maternal dietary composition during pregnancy likely affects placental nutrient delivery to the fetus and thus warrants further exploration. Similarly, maternal energy balance is an important consideration. We propose that future work in humans should consider controlling for maternal energy balance, including precise quantification of caloric intake (quality and quantity of diet using diet record analysis), and directly measured physical activity (using accelerometers), so as not to confound the results and make appropriate recommendations based on properly phenotyped subjects. Indeed, Lewis* et al.* reported lower System A activity in women with lower arm muscle mass and those who reported strenuous exercise during pregnancy [153]. Equally important is gestational weight gain, a known contributor to fetal growth [154]. The total and rate of gestational weight gain should be accounted for in all work in which fetal growth is an important outcome as excessive gestational weight gain may have a greater influence on birth weight than an underlying pathology (*i.e.*, obesity) [155]. Moreover, the body composition of both mother and infant, and how it pertains to the outcomes of interest, should be considered. Overall, if maternal dietary intake, hormones/growth factors, physical activity and gestational weight gain are associated with fetal growth parameters all future human experimental trials examining placenta transport should undertake due diligence and best account for these confounders.

Overall, a better understanding of how the placenta responds to the altered maternal milieu will be especially important in today’s transformed obesogenic environment, which includes a rise in maternal obesity, poor dietary quality, a lack of physical activity and the propensity for women to gain more than the recommended gestational weight gain independent of pregravid BMI. Further knowledge in this area may be the first step in the development of targeted interventions to help optimize fetal growth.
